# Above-ground tree carbon storage in response to nitrogen deposition in the U.S. is heterogeneous and may have weakened

**DOI:** 10.1038/s43247-023-00677-w

**Published:** 2023-02-14

**Authors:** Christopher M. Clark, R. Quinn Thomas, Kevin J. Horn

**Affiliations:** 1 U.S. Environmental Protection Agency, Office of Research and Development, Washington, DC, USA.; 2 Department of Forest Resources and Environmental Conservation, Virginia Tech, Blacksburg, VA, USA.; 3 Department of Biological Sciences, Virginia Tech, Blacksburg, VA, USA.; 4 Present address: Freedom Consulting Group, 7061 Columbia Gateway Drive, Columbia, MD, USA.

## Abstract

Changes in nitrogen (N) availability affect the ability for forest ecosystems to store carbon (C). Here we extend an analysis of the growth and survival of 94 tree species and 1.2 million trees, to estimate the incremental effects of N deposition on changes in aboveground C (dC/dN) across the contiguous U.S. (CONUS). We find that although the average effect of N deposition on aboveground C is positive for the CONUS (dC/dN=+9 kg C per kg N), there is wide variation among species and regions. Furthermore, in the Northeastern U.S. where we may compare responses from 2000–2016 with those from the 1980s–90s, we find the recent estimate of dC/dN is weaker than from the 1980s–90s due to species-level changes in responses to N deposition. This suggests that the U.S. forest C-sink varies widely across forests and may be weakening overall, possibly necessitating more aggressive climate policies than originally thought.

The effect of elevated atmospheric nitrogen (N) deposition on the terrestrial forest carbon (C) sink is an important factor affecting forest health and productivity and plays a key role in the global C cycle and any potential mitigating effect from climate change^1–3^. Atmospheric deposition of nitrogen (N) can impact forests through a variety of mechanisms^4,5^, including fertilization^6,7^, acidification (mostly from sulfur but also N)^8,9^, and from changes in foliar nutrient content which can impact pest pressures^10^. All of these may affect tree demographics by altering the growth and survival rates of trees and ultimately affect rates of forest carbon uptake. A wide range of estimates of enhanced forest C uptake with elevated atmospheric N deposition have been published using plot-level N addition experiments and analyses of forest dynamics across deposition gradients^1,2,6,11–13^. This range of estimates suggests poorly understood complexity in climatic, edaphic, biotic, temporal, or other factors that control forest responses to N. Among these complexities are the idiosyncratic responses of individual species to N deposition^6,14–17^. Most studies exploring these responses, however, have been performed at smaller scales with limited numbers of tree species and/or environmental conditions analyzed. Furthermore, studies in the U.S. have focused primarily on N deposition without explicitly considering the potential interactions or conflation with S deposition. Both N and S deposition have declined in the eastern U.S. in the past few decades^18,19^, which spatially covary due to a common source in many non-agricultural areas (i.e., fossil fuel combustion). Assessing the tree C response to N deposition while accounting for S will enable the assessment of the varying responses to N deposition among species and thus regions. This will support a better understanding of how the strength of the terrestrial C sink varies across the landscape and may be changing through time and as air quality improves.

Here we extend a recent analysis that assessed the growth and survival responses of 94 tree species across the CONUS to N and S deposition^20^ to estimate the sensitivity of aboveground C storage to N deposition (dC/dN) over 2000–16 (Fig. 1 and Methods). We then compare these findings to an earlier study based on growth rates from the 1980s–90s^6^ using similar techniques to assess whether dC/dN has changed over time at the species and forest-stand level. This analysis uses the US Forest Service Forest Inventory and Analysis (FIA) database to assess separately by species the response to N and S deposition while accounting for tree size, temperature, precipitation, and competition in the forest canopy for light. Here we focus on the associations with N deposition, because while S deposition is low and declining across much of the U.S., N deposition is still elevated across much of the U.S. relative to pre-industrial levels under which these species and ecosystems evolved, and can have a variety of relationships due to N deposition’s capacity for fertilization and acidification^21^.

## Results and Discussion

### Sensitivity of carbon storage to N deposition across the CONUS.

**Fig. 2:**
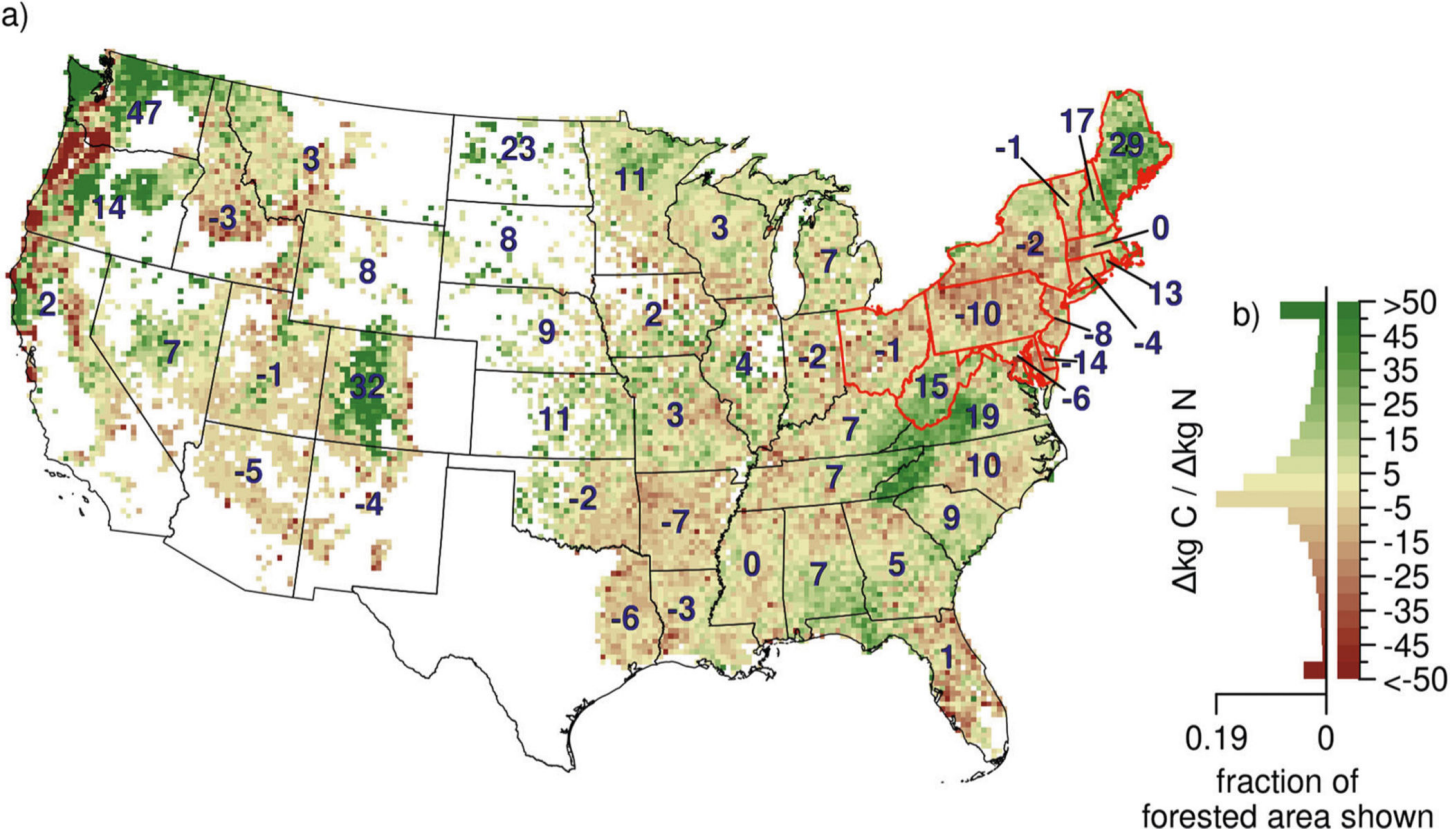
Estimates of dC/dN for the CONUS. Shown are the expanded estimates of dC/dN for trees sampled over 2000–2016 within each 20 ×20 km grid cell (**a**). State numbers indicate the summed dC/dN for increases in N deposition to forested areas within each state. States outlined in red are the same 13 states used in^6^ for trees sampled in the 1980s and 1990s. The histogram (**b**) indicates the distribution among grid cells of the changes in aboveground C to N deposition (black line for reference at the CONUS average +9 kg C kg N^−1^).

We found wide variation in dC/dN across forested regions in the U.S. (Fig. 2a), with average decreases across grid cells of more than −50 kg C kg N^−1^, or increases of more than +50 kg C kg N^−1^, and across states from −14 kg C kg N^−1^ (Delaware) to +47 kg C kg N^−1^ (Washington, Fig. 2b). Averaged across CONUS, the net change was +9 kg C kg N^−1^ (Fig. 2b). The average magnitude of increase across the CONUS is comparable to other estimates, including from a global synthesis of N fertilization studies across 41 temperate forests that found an average increase of +12.7 kg C kg N^−1 13^, from an associated meta-regression that found an increase of 0–8 kg C kg N^−1 22^, and from an observational analysis across a European N deposition gradient which found an average increase of +19 kg C kg N^−1 23^. The global study in^13^ found larger effects from N fertilization in boreal forests and nonsignificant effects in tropical forests. None of the four species examined in a recent European study^23^ were abundant enough to be included in our study, and other studies often report results at forest types (e.g., boreal, temperate) rather than species making direct comparisons difficult^13,22^. Nevertheless, a wide range among species and regions in our study was also found in^23^, which reported an increase in aboveground growth for Norway spruce (*Picea abies*) and Scots pine (*Pinus sylvestris*), and no statistically significant effect for common beech (*Fagus sylvatica*) and a combined sessile/common oak (*Quercus petraea/Quercus robur*).

The wide range of responses within and among regions was likely driven by differences in forest composition, N deposition, and species responses to N deposition. Our earlier study^20^ found wide variation among species responses to N deposition, and thus as forest composition varies, so will forest dC/dN. For example, strong increases in dC/dN (Fig. 2a) were observed in many areas and likely caused by local abundance of different species with strong positive responses - in Maine driven by strong increases of the dominant species red maple (*Acer rubrum*), in West Virginia, Virginia, and North Carolina driven by several species including yellow poplar (*Liriodendron tulipifera*), and in Colorado and Washington by Douglas fir (*Pseudotsuga menziesi*). The global meta-analysis in^13^ found stronger dC/dN effects in more northerly biomes and with lower rates of N addition (<30 kg N ha^−1^ yr^−1^) or N deposition (<15 kg N ha^−1^ yr^−1^). This is consistent with our results from the Northeast but not from our results of the Southeast, both of which were strongly positive. Patterns in the Southeast could be due to other important factors that were not included in the global meta-analysis or here (e.g., mycorrhizal association^24^). Strong decreases in our study were also observed such as in portions of Oregon which were likely driven by negatively responding species such as Engelmann spruce (*Picea engelmannii*). Additional analyses are needed for definitive attribution to species due to occasional contrasting responses from growth versus survival (e.g., for Engelmann spruce, growth decreases but survival increases with N deposition), potential correlations with other factors, and the high number of species in these forests. Regardless, variation in species sensitivity to N deposition and in forest composition means that different regions and U.S. states have different changes in their C sink with increased or decreased N, with Delaware losing C as N increases (−14 kg C kg N^−1^), and Washington gaining C (+47 kg C kg N^−1^). Even within states there could be wide variation, including decreases in the Coastal and Cascade ranges of Oregon and increases in the state’s eastern mountains and plateaus (Fig. 2a). An opposite sign for dC/dN is expected when N is decreasing.

### Comparison of dC/dN in 2000–2016 with 1980s–90s.

**Fig. 3:**
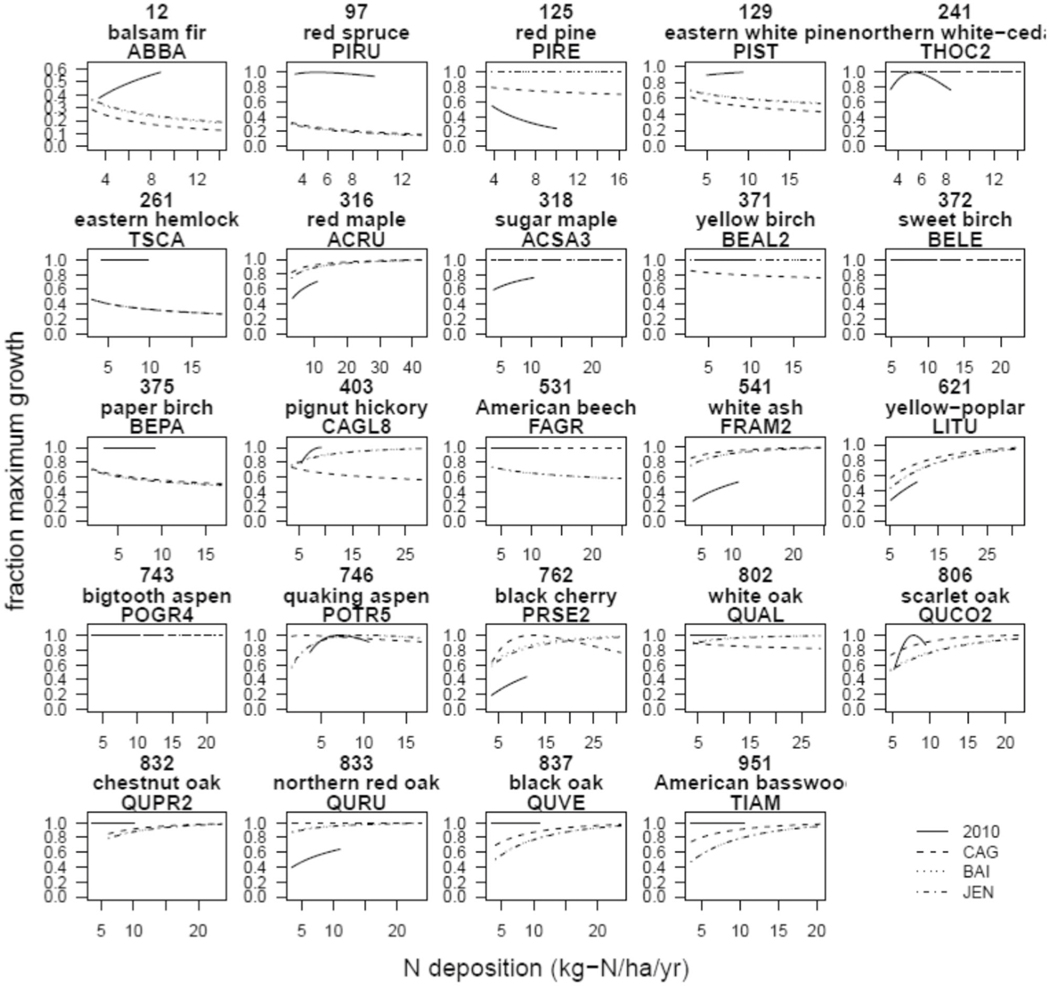
Comparison of growth responses between 2000–2016 for the CONUS (this study) and 1980s−1990s for the Northeast^6^. Each panel is a species, which includes the species common name, FIA numerical identifier, and FIA 4-letter name (e.g., *Abies balsamea* is balsam fir, 12, ABBA). Growth responses from^6^ are solid lines (“2010” in legend) while those from this study are non-solid lines. Growth responses in this study were estimated with three different methods, including the estimates of C aboveground provided by the FIA program (CAG, dashed lines), basal area index (BAI, dotted lines), and allometric responses from Jenkins et al. 2003^44^ (“JEN,” dot-dash lines). Methods in^6^ were equivalent to JEN. The y-axis is the relative growth rate normalized for a species while the x-axis is N deposition (in kg N ha^−1^ yr^−1^).

**Fig. 4:**
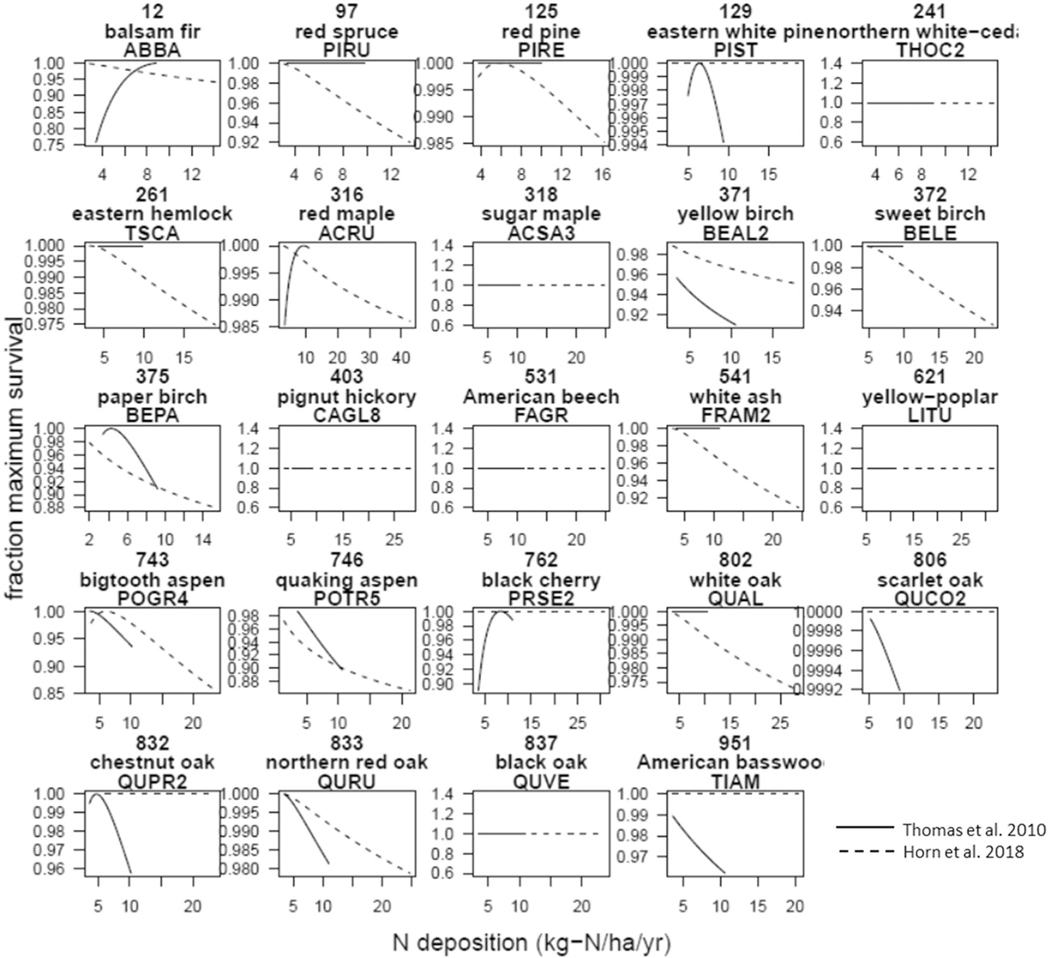
Comparison of survival responses between 2000–2016 (this study) and 1980s–1990s for the Northeast^6^. Each panel is a species, which includes the species common name, FIA identifier, and FIA 4-letter name (e.g., *Abies balsamea* is balsam fir, 12, ABBA). Responses from^6^ are solid lines (“Thomas et al. 2010” in legend) with those from this study are dashed lines (“Horn et al. 2018”). The y-axis is the relative survival rate normalized for a species while the x-axis is N deposition (in kg N ha^−1^ yr^−1^).

Our continental scale mean value was substantially lower than a previous estimate (i.e., 61 kg C kg N^−1^ from^6^) which used a similar approach but focused on only the N deposition effect in the northeastern U.S. during the 1980s and 90s. If we restrict our analysis to the same 13 state region as in^6^, our estimate (i.e., +6.7 kg C kg N^−1^) is still substantially lower than the previous value. Replicating the same analysis from^6^ (i.e., Table 2 in^6^) revealed that nitrogen deposition was no longer an important predictor for total stand-level C uptake in this region. This finding agrees with a recent multi-state, five-year, N-by-phosphorus (P) fertilization study in the northeastern U.S. that found growth increased with P but not N addition^25^. The difference between our estimate and^6^ may suggest that the contribution of N deposition to the stand-level C sink of the northeastern U.S. has diminished or disappeared over time, even though species effects remain. This could occur either through continued N saturation^26,27^, forest aging^22^, accumulated effects from historical S deposition limiting current forest responses^28^, nutrient limitation currently by something other than N (e.g., P^25^), or some combination of these effects. Lower N deposition does not appear to explain these results as the fundamental shape of the relationships has changed (Figs. 3 and 4, next section) even for similar ranges of deposition. Our results, though consistent with other studies^13,22,23^, also suggests that a static estimate of dC/dN for temperature forests may not be reliable over longer intervals. This may be true for other forest types as well. Although not definitive, our findings combined with^25^ suggests that long-term deposition of N may not provide sustained stimulation of the C cycle. A weakening of this response has implications on the degree of climate policies needed to combat climate change.

### Comparison of species-level responses in 2000–2016 with 1980s–90s.

We next explored which species responses to N deposition may have changed between the 1980s–90s and 2000–2016. Direct comparison of the 24 species shared across the two studies revealed only one species whose growth (Fig. 3, Supplementary Table 1) and survival (Fig. 4, Supplementary Table 1) relationships with N deposition were relatively unchanged between the 80s–90s and 2000–2016 (*Liriodendron tulipifera*). There were eight species for which the growth responses had become more negative between 1980s–90s and 2000–2016, seven for which the survival responses had become more negative, and three for which both had become more negative (*Abies balsamea, Picea rubens, Tsuga canadensis*; Supplementary Table 1). For example, *Abies balsamea*, a common species in the Northeast, had switched from a positive relationship in the 1980s–90s between N deposition and growth or survival, to a negative relationship for both, regardless of how carbon was estimated for growth (Figs. 3, 4, Supplementary Table 1). On the positive side, there were five and three species whose growth or survival relationships, respectively, had become more positive, and two for which both responses had become more positive (*Quercus coccinea, Tilia americana*, Supplementary Table 1). Thus, the relationship between N deposition and key tree responses has changed over time, and more changed in a negative direction than positive. Direct comparison between these estimates is challenging since the analytical approach differed slightly, but a more negative response here for several species is consistent with our finding of a weakened forest C sink. Further study is needed to understand why species’ responses are changing in different directions. Seven of the eight species with worsening growth responses to N deposition formed predominantly ectomycorrhizal associations (Supplementary Table 1), supporting a dominant effect from mycorrhizal association as found elsewhere^24,29^; however, five of the six species with improving relationships were also ectomycorrhizal, suggesting that other factors such as those examined in refs. ^13,22^ or phylogeny (four were oaks) may also be important. A detrimental effect from ectomycorrhizal associations due to competition with nearby AMF-trees should only manifest in mixed species x mycorrhizal stands. For example, in a stand where tree species share a common mycorrhizal association (e.g., all AMF), there may be no competitive advantage from the mycorrhizal association among tree species. Additional tree range overlap analyses with mycorrhizal associations may help reveal the prevalence of this effect and/or incorporating the range of mycorrhizal functions even within these broad classes^30,31^.

Though the incremental effect of increasing N deposition in the CONUS was found to increase aboveground C on average (Fig. 2b), N deposition is not increasing across much of the U.S. as a result of successful air quality policies. Total N deposition has decreased across much of the forested eastern U.S. and is unchanged across much of the forested western U.S.^32,33^. This has occurred from decreases in oxidized N^19^, while reduced N is increasing or unchanged^34^. This has implications for the U.S. forest C sink, which may increase in some areas like Pennsylvania and Delaware (areas with negative dC/dN, Fig. 2a), but we project these increases will be offset by a larger weakening of the forest C sink elsewhere (areas with positive dC/dN, Fig. 2a).

### Uncertainties, Implications, and Next Steps.

There are several important uncertainties in our study that deserve discussion and improvement. First, there are many factors that affect forest tree growth and survival that are not included in the present study. P deposition is increasingly important in some areas of the U.S., especially the Northeast^25^, and it may be important to include this potential driver into future models. The best source for atmospheric deposition estimates in the U.S.^32^ does not currently include phosphorus, as it is not included in the underlying monitoring network and only globally modeled estimates are available^35^ that cover the entire CONUS to our knowledge. Ozone also affects tree species growth and survival^36^, and although much less is known about sensitivity to adult trees compared with seedlings or saplings in fumigation studies^37^, this factor could be included in a similar manner as N and S deposition. CO_2_ concentrations also affect tree growth^38^, although this factor likely would need a longer temporal record than ours to detect due to lower spatial variability in atmospheric CO_2_ that prevents space for-time analysis. However, recent empirical evidence from Canada suggests that the effect from CO_2_ may be much weaker compared with strong effects from temperature and soil water^39^. This analysis of plot level tree growth across Canada did not find a significant effect CO_2_ over a 50 year record, and none of the 19 species examined had a positive individual effect^39^. Second, many of the factors that are included in our study could be imperfect surrogates for more direct causal effects. Mean annual temperature (MAT) and mean annual precipitation (MAP) in our study are surrogate variables for many factors that more directly affect tree growth and survival. For example, there are many more nuanced temperature and temperature-related effects that could be included (e.g., summer temperature, winter temperature, day vs. nighttime temperature, number of degree days above some threshold, vapor pressure deficit [VPD], etc.). However, these are all likely correlated with MAT, and given that each plot is remeasured only once every few years (average of 8.2 year remeasurement period), it would be challenging to disentangle these more nuanced temperature effects using a approach like ours. The same is true for precipitation, where many additional variables are likely more mechanistically linked (e.g., soil water, drought, effect of CO_2_), but as with temperature our study is not able to resolve these responses. In this first examination, we intentionally focused on macro-ecological factors as we are not yet able to resolve these finer details. More detailed field campaigns (e.g.,^40^), that are often at one or a few sites, are needed to resolve these mechanistic linkages. Soil factors related to site fertility also are related to responsiveness to N deposition^22^, which are poorly represented by MAT and TAP, but there are no reliable soil N estimates available for the CONUS to our knowledge. Many of these factors that are not included or are poorly represented would not affect our results provided the omitted variables are not spatially correlated with factors that are included. Additional analyses of these potentially covarying factors and others are needed. Nonetheless, several of these factors are being incorporated into an ongoing improvement of these models, as the results become available these estimates of dC/dN for the CONUS will be updated.

## Conclusions

Despite the limitations discussed above, this study has important implications. A weakening responsiveness of the U.S. forest C sink to N deposition may aggravate effects from climate change, but the solution is not to emit more N. N deposition has a host of negative effects, including contributing to soil acidification, losses of biodiversity, and nutrient pollution to waterways^41,42^. The solution is to continue to clean up the air, but to realize that this may reveal unanticipated side-effects. Furthermore, the increased C-sequestration from simultaneous reductions in S deposition could offset the reductions due to declining N deposition. These multi-pollutant dynamics are rarely assessed and require further study. At its core, the differences among regions reported here appear to be mainly a result of species-specific differences in response and local variation in N deposition and forest composition. We are only beginning to understand what drives this variation among species and regions, which are likely a result of many factors, including variation in mycorrhizal associations^24,29,30^, climate^13,22^, differential ability among species to respond to increasing levels of N^20^, soil and stand factors including forest age^13,22^, and recovery from acid deposition of N and S^28,43^. Separating the contributions from these factors, as well as inclusion of the various factors omitted or imperfectly represented, would constitute an important step forward in understanding forest responses to global change and inform potential management interventions.

## Methods

### Forest Inventory data.

Tree growth, tree survival, and plot-level basal area data were compiled from the Forest Inventory and Analysis (FIA) program database (accessed on January 24, 2017, FIA phase 2 manual version 6.1; http://www.fia.fs.fed.us/). Aboveground tree biomass was estimated from tree diameter measurements^44^ and then multiplied by 0.5 to estimate aboveground C. Tree growth rates were calculated from the difference in estimated aboveground C between the latest and first live measurement of every tree and divided by the elapsed time between measurements to the day. Tree species that had at least 2000 individual trees after the data filters were applied were retained for further growth and survival evaluation. The probability of tree survival was calculated using the first measurement to the last measurement of a plot. Trees that were alive at both measurements were assigned a value of 1 (survived) and trees alive at the first and dead at the last measurement were assigned a value of 0 (dead). The duration between the first and last measurement was used to determine the annual probability of tree survival. Trees that were recorded as dead at both measurement inventories and trees that were harvested were excluded from the survival analysis.

### Predictor data: Climate, deposition, size, and competition.

There were six predictors that were related to the response rate of growth or survival for each individual tree: mean annual temperature, mean annual precipitation, mean annual total nitrogen deposition, mean annual total S deposition, tree size, and plot-level competition.

To obtain total N and S deposition rates for each tree, we used spatially modeled N and S deposition data from the National Atmospheric Deposition Program’s Total Deposition Science Committee^32^. Annual N and S deposition rates were then averaged from the first year of measurement to the last year of measurement for every tree so that each tree had an individualized average N deposition based on the remeasurement years, and each species had an individualized range of average N deposition exposure based on its distribution. Monthly mean temperature and precipitation values were obtained in a gridded (4 × 4 km) format from the PRISM Climate Group at Oregon State^45^ for the contiguous US and averaged between measurement periods for each tree in a similar manner. Tree size was represented by estimated aboveground tree C (previously described). Because the climate and deposition predictors were tailored to each plot, the years assessed varied by plot, but spanned 2000–2016. Thus, the results from the earlier study^6^ used conditions from the 1980–1990s, whereas the results from this study used more recent environmental and stand conditions. Tree competition was represented by a combination two factors: (1) plot basal area and (2) the basal area of trees larger than the focal tree being modeled. How all six variables were statistically modeled is discussed below.

### Modeling tree growth and survival.

We developed in ref. ^20^ multiple models to predict tree growth (G; kg C year^−1^) and survival (P(s); annual probability of survival). Our growth model (Eq. 1 and 2) assumes that there is a potential maximum growth rate (a) that is modified by up to six predictors in our study (which are multipliers from 0 to 1): temperature (T), precipitation (P), N deposition (N), S deposition (S), tree size (m), and competition. The potential full growth model included all six terms (Eq. 1 for the general form and Eq. 2 for the specific form). The size effect was modeled as a power function (z) based on the aboveground biomass (m). N deposition may affect the allometric relationships between tree diameter and aboveground tree biomass^46^, but these relationships are not yet accounted for in U.S. inventories^44^. Competition between trees was modeled as a function of plot basal area (BA) and the basal area of trees larger than that of the tree of interest (BAL) similar to the methods of^47^. The environmental factors (N deposition, S deposition, temperature, precipitation) were modeled as two-term lognormal functions (e.g., $t_{1}$ and $t_{2}$ for temperature effects, *n*_1_ and *n*_2_ for nitrogen deposition effects). The two-term lognormal functions allowed for flexibility in both the location of the peak (determined by $t_{1}$ for temperature, for example), and the steepness of the curve (determined by $t_{2}$ for temperature, for example). Thus, the full growth model is presented in Eq. 2.


(1) $$G=potentialgrowthrate\times competition\times temperature\times precipitation\times S_{dep}\times N_{dep}$$

(2) $$G=a*m^{z}*e^{\left(c_{1}*BAL+c_{2}*\ln(BA)\right)}*e^{-0.5*{\left(\frac{ln\left(T/t_{1}\right)}{t_{2}}\right)}^{2}}*e^{-0.5*{\left(\frac{ln\left(P/p_{1}\right)}{P_{2}}\right)}^{2}}*e^{-0.5*{\left(\frac{ln\left(N/n_{1}\right)}{n_{2}}\right)}^{2}}*e^{-0.5*{\left(\frac{ln\left(S/s_{1}\right)}{s_{2}}\right)}^{2}}$$

We examined a total of five different growth models: (1) a full model with the size, competition, climate, S deposition, and N deposition terms (Eq. 2); (2) a model with all terms except the N deposition term; (3) a model with all terms except the S deposition term; (4) a model with all terms but without S and N deposition terms; and (5) a null model that estimated a single parameter for the mean growth parameter (*a* in Eq. 2).

The annual probability of survival (P(s)) was estimated similarly as for growth, except that the probability was a function of time and we explored two different representations for competition. The general form of the model is shown in Eq. 3, and the full survival model in Eqs. 4, 5 for the two competition forms.


(3) $$P(s)={\left[a\cdot \text{size}\times competition\times temperature\times precipitation\times N_{dep}\times S_{dep}\right]}^{time}$$

(4) $$P(s)={[a*\left[\left(\left(1-zc_{1}e^{-zc_{2}*m}\right)*e^{-zc_{3}*m^{zc_{4}}}\right)\left(e^{-br_{1}*{BA_{\text{ratio}}}^{br_{2}}*BA^{br_{3}}}\right)\right]}^{time}\;\;{*e^{-0.5*{\left(\frac{ln\left(T/t_{1}\right)}{t_{2}}\right)}^{2}}*e^{-0.5*{\left(\frac{ln\left(P/p_{1}\right)}{p_{2}}\right)}^{2}}*e^{-0.5*{\left(\frac{ln\left(N/n_{1}\right)}{n_{2}}\right)}^{2}}*e^{-0.5*{\left(\frac{ln\left(S/s_{1}\right)}{s_{2}}\right)}^{2}}]}^{time}$$

(5) $$P(s)=[a*\left(e^{-0.5*{\left(\frac{(ln\left(m/m_{1}\right)}{m_{2}}\right)}^{2}*-0.5*{\left(\frac{ln\left(BA/ba_{1}\right)}{ba_{2}}\right)}^{2}*-0.5*{\left(\frac{ln\left(BAL+1/bl_{1}+1\right)}{bl_{2}}\right)}^{2}}\right)\;\;{*e^{-0.5*{\left(\frac{ln\left(T/t_{1}\right)}{t_{2}}\right)}^{2}}*e^{-0.5*{\left(\frac{ln\left(P/p_{1}\right)}{p_{2}}\right)}^{2}}*e^{-0.5*{\left(\frac{ln\left(N/n_{1}\right)}{n_{2}}\right)}^{2}}*e^{-0.5*{\left(\frac{ln\left(S/s_{1}\right)}{s_{2}}\right)}^{2}}]}^{time}$$

A total of nine survival models were examined: four using the formulation for size and competition in Eq. 4 (with the same combinations of predictors as above for growth), four using formulation for size and competition in Eq. 5, and a null survival model in which a mean annual estimate of survival (*a*) was raised to the exponent of the elapsed time.

Parameters for each of the growth and survival models above were fit for a given species using maximum likelihood estimates through simulated annealing with 100,000 iterations via the likelihood package (v2.1.1) in Program R. Akaike’s Information Criteria (AIC) was estimated for all models. The best model was the model with the lowest AIC, and statistically indistinguishable models are those with a delta AIC < 2^48^. We used the simplest model (i.e., the one with the fewest parameters) among the set of statistically indistinguishable models as the basis for dC/dN. The variation explained in the models in ref. ^20^ was good for growth (R^2^ averaged 24% for the 94 species +/− 15% standard deviation) and was not reported for survival. Additional details can be found in^20^.

### Estimating dC/dN from individual responses from N and S deposition.

Individual tree growth and survival equations were combined to estimate the relationships between N deposition and aboveground tree C calculated as the change in aboveground tree C accumulation vs change in N deposition rates ($\text{Δ}\;{\text{kg C ha}}^{-1}{\text{yr}}^{-1}/\text{Δ}\;{\text{kg N ha}}^{-1}\;{\text{yr}}^{-1}$ or dC/dN). The estimated amount a tree grows after 1 year is simply its annual growth rate (G). The estimated amount of aboveground tree biomass for a single surviving tree that carries over from one time period to the next is the initial biomass plus the growth (Eq. 6).


(6) $$\text{Biomass of surviving tree}=G+C_{i}$$

For the FIA database, at the landscape level the amount of forest biomass associated with a tree is the amount of biomass associated with that tree (Eq. 6), multiplied by the FIA expansion factor (f in Eq. 7; because the FIA tree represents many trees), multiplied by the probability of survival (P(s) in Eq. 7, because not all expanded trees will survive).


(7) $$\text{Landscape level forest biomass from surviving trees}=\left(G+C_{i}\right)\cdot f\cdot P(s)$$

(8) $$\text{Plot level forest biomass from surviving trees}=\left(G+C_{i}\right)\cdot P(s)$$

At the plot level, the expansion factor is not needed and the equation for the amount of tree biomass simplifies to (Eq. 8). The annual aboveground tree C accumulation, for established trees, is the difference in the initial aboveground tree $\text{C}\left({\text{C}}_{\text{i}}\right)$ and the aboveground tree C after 1 year in $\text{kg}\;\text{C}\;{\text{tree}}^{-1}\;{\text{yr}}^{-1}$ (Eq. 9).


(9) $$\text{Annual aboveground tree}\;C\;\text{accumulation}=\left(G+C_{i}\right)\cdot P(s)-C_{i}$$

We estimated the rate of change in aboveground tree C accumulation vs N deposition (dC/dN) by estimating the annual aboveground tree C accumulation (Eq. 9) at the local rate of N deposition and the local rate of N deposition plus 0.01 kg N ha^−1^yr^−1^, allowing us numerically calculate the slope of the relationship between C accumulation and N deposition. Subtracting these two calculations of annual C accumulation provides the change in C associated with a small change in N (dC). We normalized this calculation by dividing the small change in N deposition (0.01 kg N ha^−1^ yr^−1^) to estimate dC/dN for each tree within a plot. To scale the individual tree estimates to the plot level, individual tree (dC/dN) values were summed up by FIA plot and divided by the plot area. To scale from the plot level to the landscape, plot dC/dN values were then averaged within each 20 × 20 km pixel and weighted by its corresponding plot expansion factor (see FIA manual).

### Comparison of responses in 2000–2016 with 1980s–90s.

The relationships from the study focused on the 1980s–90s^6^ did not include sulfur deposition, thus, those relationships are better described as the effect from N deposition when only N is included in the model (i.e., does not try to account for S deposition). In the study focused on 2000–2016^20^ we ran models with only N, with only S, and with both. Thus, to optimize the comparison with the earlier study we selected the model in^20^ that only included N, regardless of whether it was the best overall model. These comparisons are shown in Figs. 3, 4. N and S were only weakly associated in much of this region (Fig. 5 from ref. ^20^). Also note, we examined three different allometric scaling methods estimating C from FIA diameter measurements (CAG, BAI, and Jenkins). The most appropriate comparison with^6^ is Jenkins, while the others were included to examine sensitivity to the method for future work. To further compare to the estimates of dC/dN from 1980–90s in ref. ^6^, we ran a second analysis using stand-level C increment between two measurements periods (rather than the up-scaled species response curves described above) to assess the relationship between N deposition on annual C increment of all surviving trees in a plot (i.e., as in Table 2 in ref. ^6^). Following^6^, we first ran a model with climate and size (i.e., carbon stock at the first measurement period) terms. Then we added N deposition to that model to see if the AIC value decreased by 2 or more. Although the addition of N deposition led to a significant improvement of the stand-level model in ref. ^6^ in the Northeastern U.S. for the 1980s–90s, it did not in this analysis of the 2000–2016 period.

## Supplementary Material

SI

## Figures and Tables

**Fig. 1:**
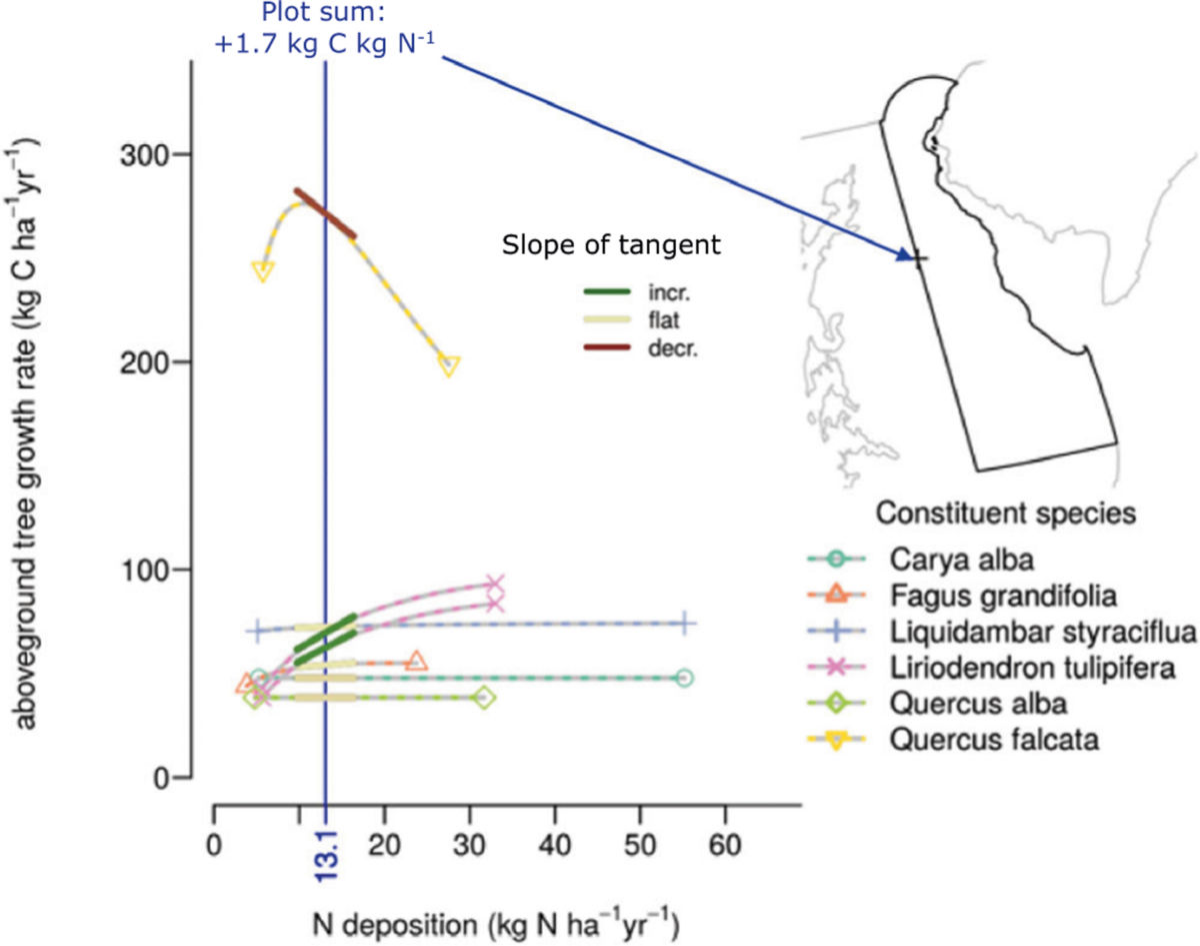
A schematic illustrating how plot-level dC/dN rates were calculated. The example plot is located in eastern Delaware, U.S., and has seven trees representing six different species. The individual tree-level curves differ for the same species based on the tree’s size and location in the canopy. Individual slopes are estimated at the local N deposition rate (13.1 kg N ha^−1^ yr^−1^), which are then summed to get a plot-level instantaneous estimate of C accrual with an increase in N deposition (example plot: growth increases with N deposition by 1.7 kg C kg N^−1^). In this example, the growth increase for the two yellow poplar trees (*Lirodendron tulipifera*) offsets the decrease from the one southern red oak tree (*Quercus falcata*), with the other species being relatively unaffected. A similar approach was used to calculate the responsiveness of plot-level survival to N deposition.

## Data Availability

Data for this study are available in the EPA’s Environmental Dataset Gateway at https://edg.epa.gov/ (https://doi.org/10.23719/1528045).
