# Supplementary material for: Above-ground tree carbon storage in response to nitrogen deposition in the U.S. is heterogeneous and may have weakened
Source: Commun Earth Environ. Author manuscript; Available in PMC 2024 Feb 14. (PMC10262689; doi:10.1038/s43247-023-00677-w)
Supplement: SI [file NIHMS1879583-supplement-SI.docx]

**Supplementary Information.**

**Supplementary Table 1: Summary of the change in responses between [1] and this study (i.e., summary of Figures 3 and 4 in the main text).** Shown below are whether the N response for growth or survival from the 80s-90s [1] and from 2000-2016 (this study) was either positive (+), negative (-), flat (0), or unimodal (∩). Only the Jenkins estimation method is included here since that is most comparable to [1]. Relationships that became more negative (orange), more positive (green), or were unchanged (blue) or uncertain (white) are also shown. There are several ways a relationship could become more negative (e.g., from + to -, from 0 to -, etc.), or more positive. Also shown is whether the species forms dominant mycorrhizal associations with arbuscular mycorrhizae (AM), ectomycorrhizae (ECM), or either (Either) from [2]. See Figures 3 and 4 for more details on the individual changes summarized here.

| Common name | AM/  ECM | Growth | | Survival | |
| --- | --- | --- | --- | --- | --- |
|  |  | 80s-90s | 2000-2014 | 80s-90s | 2000-2014 |
| Balsam fir | ECM | + | - | + | - |
| Red spruce | ECM | 0 | - | 0 | - |
| Red pine | ECM | - | - | 0 | ∩ |
| Eastern white pine | ECM | + | - | ∩ | 0 |
| Northern white cedar | AM | ∩ | 0 | 0 | 0 |
| Eastern hemlock | ECM | 0 | - | 0 | - |
| Red maple | AM | + | + | + | - |
| Sugar maple | AM | + | 0 | 0 | 0 |
| Yellow birch | ECM | 0 | - | - | - |
| Sweet birch | ECM | 0 | 0 | 0 | - |
| Paper birch | ECM | 0 | - | - | - |
| Pignut hickory | ECM | + | + | 0 | 0 |
| American beech | ECM | 0 | - | 0 | 0 |
| White ash | AM | + | + | 0 | - |
| Yellow-poplar | AM | + | + | 0 | 0 |
| Bigtooth aspen | Either | 0 | 0 | - | ∩ |
| Quaking aspen | Either | ∩ | ∩ | - | - |
| Black cherry | AM | + | + | ∩ | 0 |
| White oak | ECM | 0 | + | 0 | - |
| Scarlet oak | ECM | ∩ | + | - | 0 |
| Chestnut oak | ECM | 0 | + | ∩ | 0 |
| Northern red oak | ECM | + | + | - | - |
| Black oak | ECM | 0 | + | 0 | 0 |
| American basswood | ECM | 0 | + | - | 0 |

**Supplementary References**

1. Thomas, R.Q., et al., *Increased tree carbon storage in response to nitrogen deposition in the US.* Nature Geoscience, 2010. **3**(1): p. 13-17.

2. Phillips, R.P., E. Brzostek, and M.G. Midgley, *The mycorrhizal‐associated nutrient economy: a new framework for predicting carbon–nutrient couplings in temperate forests.* New Phytologist, 2013. **199**(1): p. 41-51.
